# Displacement Calculation for Service Loads of Reinforced Concrete Beams and Slabs Using Physically Non-Linear Analysis

**DOI:** 10.3390/ma15238307

**Published:** 2022-11-23

**Authors:** Antonio Renato Bicelli, Pedro Cantor, Rafael Wong, Mario Rui Arruda

**Affiliations:** 1 Instituto Superior Técnico, Universidade de Lisboa, 1049-001 Lisbon, Portugal; 2 Faculdade de Ciências e Tecnologia, Universidade Nova de Lisboa (UNL), 2825-149 Caparica, Portugal; 3 CERIS—Instituto Superior Técnico, 1049-001 Lisbon, Portugal

**Keywords:** physically non-linear analysis, cracked state, multi-layer method, displacement calculation

## Abstract

This paper aims to use non-linear physical analysis to calculate the displacement of beams and slabs in a cracked state. This study uses the commercial software SAP2000 to perform a numerical analysis using the finite element formulation, applying the multi-layer method. Initially, a parametric study was carried out to evaluate the vertical displacement for service loads of reinforced concrete beams and slabs using different spans, support conditions and geometry. In order to validate the finite element model, the study compared displacement values for linear analysis using Bares tables. Subsequently, simplified methods of displacement calculation in the long term are applied, using an abacus from *Comité Euro-International du Béton* (CEB). These values are then compared with the physically non-linear analysis in the long-term cracked state with SAP2000. Two structural codes were used in the numerical and analytical methods, *Regulamento de Estruturas de Betão Armado e Pré-Esforçado* (REBAP) and Eurocode 2 (EC2), to evaluate their differences in deformation control. Therefore, the main goal is to establish comparisons between the two methods of analysis in order to show that non-linear finite element modelling provides values that are lower than the analytical calculation, thus allowing greater economy in the design of structural reinforced concrete elements. In addition, it can be noted that EC2 has some inconsistencies in the design of simply supported slabs, requiring a greater thickness than a flat square slab and that indirect deformation for REBAP is underestimated for beams and, in some cases, for slabs.

## 1. Introduction

The idea of using non-linear analysis in reinforced concrete structures to promote efficient and economic structural design is not new [1,2], but the restrictions of structural code guidelines [3] prevented the widespread use of non-linear analysis [4]. Although the use of frame elements in non-linear structural concrete design is quite popular in the scientific community [5,6], its application still leaves some uncertainties for the structural designer [7,8]; this is even more pronounced in reinforced concrete shell elements [9].

The effects on the strength of reinforced concrete (RC) beams with fiber-reinforced polymer were investigated experimentally and numerically in a precracked condition [10], showing an improvement in the ultimate load capacity of the member. Cosenza [11] developed a finite element analysis of reinforced concrete elements in the cracked state that provides an accurate methodology for evaluating the stiffness matrix and load vector. The significance of the moment of inertia in the cracked cross-section in the analysis of structural elements for service load was demonstrated in [12] by experimental testing of RC beams with glass fiber-reinforced polymer and steel-reinforced beams with different ratios of gross to cracked cross-section moment of inertia.

The control of deformation of reinforced concrete elements is of utmost importance due to the current requirements regarding excessive cracking and deformation [13,14]. In addition, there has been an increased use of elements with great height and slenderness, which requires a more rigorous deformation control. Structural codes used in Portugal for the control of deformations in reinforced concrete structures, namely *Regulamento de Estruturas de Betão Armado e Pré-Esforçado* (REBAP [15]) and Eurocode 2 (EC2 [16]), provide two types of limit states: ultimate limit state and service limit state. Although failure to meet a design criterion for deformation does not compromise the safety of structures at the failure level, it is necessary to ensure excellent behavior of structures under service loads. Furthermore, good behavior avoids inconvenience to users due to the poor esthetics of structures associated with excessive cracking, which is the main objective of the Portuguese structural code.

The allowable values for maximum displacement are listed in ISO 4356:1997, referenced by both EC2 and REBAP, and depend mainly on the dimensions of the structural elements and the type of use of the structure. These two structural codes on the control of deformations in reinforced concrete structures indicate two different ways of controlling them. In simple terms, they are the direct control and the indirect control. The first is the explicit calculation of the maximum displacement and its comparison with the maximum allowable value. On the other hand, the second considers that the use of the limit slenderness allows the exemption from the calculation of explicit deformation. That is, it can be designed at the level of the geometry of the structural elements to avoid the verification by direct control.

The present study is intended to analyze the performance of these two criteria for the control of structural deformations of reinforced concrete beams and slabs. For this purpose, structural elements are designed to verify the indirect control of the deformations for each code. Each element explicitly calculates the respective deformations and compares them with the limit values. In this way, it is possible to understand how much leeway indirect control offers compared to these limits and whether the increased difficulty at the level of deformation calculation in direct control can be compensated by assumed solutions with less effort.

The calculation is presented in two ways: an analytical study using expressions and a non-linear numerical analysis [17] using a commercial finite element software, SAP2000. The new versions of SAP2000 can simulate concrete’s non-linear behavior, including cracking inelastic strains with layered shell finite elements. The decision to use SAP2000 is because it is more related to conventional structural engineering than ABAQUS or ANSYS. The innovative contribution of the work relates to the finite element modeling of non-linear shell elements along the thickness and span for structural elements of slabs and beams in the context of the evaluation of deformations based on criteria for the design according to EC2 and REBAP. Finally, it is checked whether there is a significant difference between the deformation values obtained according to EC2 and REBAP design criteria. The applications guideline for non-linear finite element analysis in structural design are presented in the report of [18], using the reliability design from the work of [19].

## 2. Methods

The current structural codes in Portugal allow the exemption from the control of deformations if the criteria for slenderness of the structural elements are met. In this section, the criteria required to avoid direct control of deformation are given, as well as the minimum height for slabs and beams for each code. Finally, the methodology for calculating the long-term deformations is explained.

### 2.1. REBAP Slenderness

The design principles of structural elements shall be established to relieve the verification of deformation by explicit calculation according to REBAP. According to Article 72° of REBAP, this satisfies the safety of deformation limits when conditions specified in Articles 89°, 102° and 113° are met. Article 89° establishes that the following expression calculates the minimum height of the beam:

(1) $$\frac{li}{h}=20\eta$$

where *h* is beam height; *li = l* ∗ *α* is span equivalent to the beam, where *l* is the theoretical span and *α* is a coefficient depending on the support conditions and *η* is a coefficient depending on the type of steel. Article 102° specifies the value of the thickness of the slab, in which the parameters have the same denomination as before, through:

(2) $$\frac{li}{h}=30\eta$$

This study also recommends that the thickness be less than 7 cm if it is a slab subjected mainly to a distributed load, and 15cm for a slab supported directly on pillars. The parameter *α* depends on the support conditions and is given in Table XIII of Article 89° and Table XV of Article 102°, respectively, for beams and slabs. The steel used in this work is A500NR. Therefore, the parameter *η* is 0.8. Table 1 shows the slenderness limit values for indirect control corresponding to the different support conditions. In cases where the structural code did not cover this, the intermediate values between the two situations closest to the study case were used. The expression for calculating the minimum thickness of the flat slab is identical to that for the simply supported slab, since there is no specific expression for this case in REBAP (Table 1).

Article 113° establishes the theoretical span to be considered for the lightened slabs, which is not discussed in this paper. The width of the compression flange of T beams was taken into account in the design of beams. According to Gomes et al. [20], the dimensions determined with REBAP might be insufficient to verify the structural deformation criterion.

### 2.2. EC2 Slenderness

In this subsection, the design principles for structural elements are presented to allow the verification of deformation by explicit calculation (direct control) according to EC2. Expressions 7.16a and 7.16b from EC2 [16] were used to define the cross-sectional geometry of structural elements (beams and slabs) in terms of the slenderness limit value (*l/d*) sufficient to avoid verification of direct deformations.

(3) $$7.16a\;\{\frac{l}{d}=K\left[11+1,5\;\sqrt{f_{ck}}\;\frac{\rho_{0}}{\rho }+3,2\;\sqrt{f_{ck}}{\left(\frac{\rho_{0}}{\rho }-1\right)}^{\frac{3}{2}}\right]\;if\;\rho \leq \rho_{0}$$

(4) $$7.16b\{\frac{l}{d}=K\left[11+1,5\;\sqrt{f_{ck}}\;\frac{\rho_{0}}{\rho -\rho^{\prime }}+\frac{1}{12}\;\sqrt{f_{ck}}\sqrt{\frac{\rho^{\prime }}{\rho_{0}}}\right]\;\;\;\;\;\;if\;\rho >\rho_{0}$$

where: *l*—the span of beam and slab; *d*—useful height of the slab or beam, assuming the value of 3 cm for the first coating and 5cm for the second; *K*—coefficient of the different structural systems; *ρ_0_*—required reference reinforcement ratio calculated by $10^{-3}\sqrt{f_{ck}}$ (MPa); *ρ*—required tensile reinforcement ratio at middle span and *ρ’*—required compressive reinforcement ratio at middle span. The expression used to calculate the parameter *ρ* is:

(5) $$\rho =\frac{b\times d}{A_{s}}$$

where: *b*—cross-section width; *d*—cross-section useful height and *A_s_*—required tensile reinforcement at middle span. It was assumed that the beam is heavily loaded, thus *ρ* = 1.5% is required. The slabs, on the other hand, were assumed to be subjected to minor stresses, thus *ρ* = 0.5% is required. Therefore, these reinforcement levels were used in a very simplified way in the models studied, assuming that the ultimate limit states were checked. REBAP does not specify reinforcement ratios required for expressions that do not require direct control of deformation. Hence, the same reinforcement ratios were applied in EC2. The *K* values were taken from Table 7.4N in [16]. In this work, different parameterizations were used, i.e., C30/37 concrete and steel with yield stress of 310MPa, satisfying the conditions for using the values of Table 7.4N in [16].

The value used for the width of the beam was 40% of the estimated value for the height of the beam. However, according to item 5.3.2.1 of EC2, it is possible to take into account an effective width of the flanges. In this way, the value of the effective width was taken into account in the analytical inertia calculations of the beam to obtain a structural behavior closer to reality.

### 2.3. Minimum Dimensions Comparison between Structural Codes

**Figure 1:**
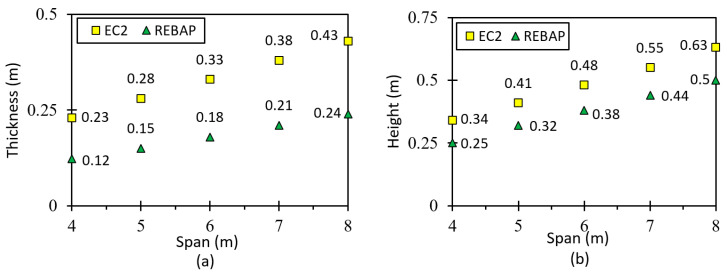
(**a**) Minimum thickness of the simply supported slab and (**b**) minimum height of the supported beam.

To verify indirect deformation control, this section gives the minimum thicknesses and heights of beams and slabs required by EC2 and REBAP, respectively, for a span varying from 4 m to 8 m. Therefore, a 5 cm and 3 cm thick coating was assumed for beams and slabs, respectively. Figure 1 shows the minimum thickness for the simply supported slab and the minimum height for the beam considering the supported square slab. Considering these diagrams together, the minimum design values that do not require direct control of structural elements are higher in EC2 than in REBAP. It can also be observed that the differences between the structural codes are greater for the slab than for the beam.

**Figure 2:**
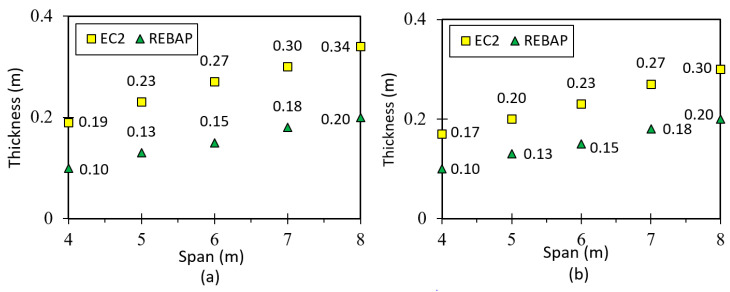
(**a**) Minimum thickness of the fixed-ended supported slab and (**b**) of the fixed-ended slab.

Figure 2a shows the minimum thickness of the slab supported at two edges and at the remaining edges with fixed-ended conditions. From Figure 2, it can be seen that the thickness according to EC2 is greater than that according to REBAP and that the differences between the thicknesses increase as the span increases. The minimum thicknesses of the fixed-ended slab on the four edges are shown in Figure 2b. As in the previous cases, the minimum thicknesses calculated according to EC2 are greater than the slab thicknesses calculated according to REBAP. It can be concluded that, regardless of the support conditions to which the slabs are subjected, greater thicknesses are required for design according to EC2 than according to REBAP.

**Figure 3:**
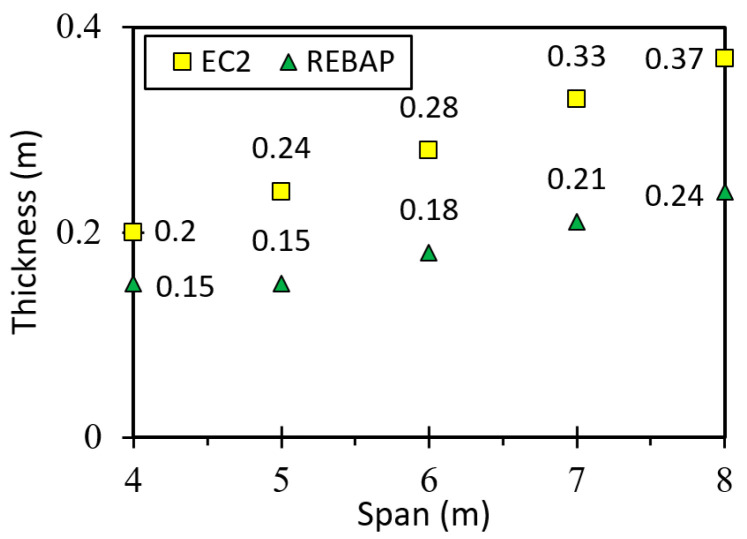
Minimum thickness of the square flat slab.

For the last case, the square flat slab, the minimum thickness is given in Figure 3. For this type of slab, there is no specific expression in REBAP, and it is necessary to use Equation (2), which applies to a solid slab. However, Equation (2) mentions the minimum thickness, which requires the same value for spans of 4 m and 5 m. Figure 3 shows that the minimum thickness value for EC2 is greater than for REBAP and that the difference between the structural codes increases as the span increases. In all cases studied, the minimum dimensions of the structural elements designed by EC2 are greater than those of REBAP. Thus, the deformation values are higher for the elements designed with REBAP.

### 2.4. Long-Term Deformation Method

**Figure 4:**
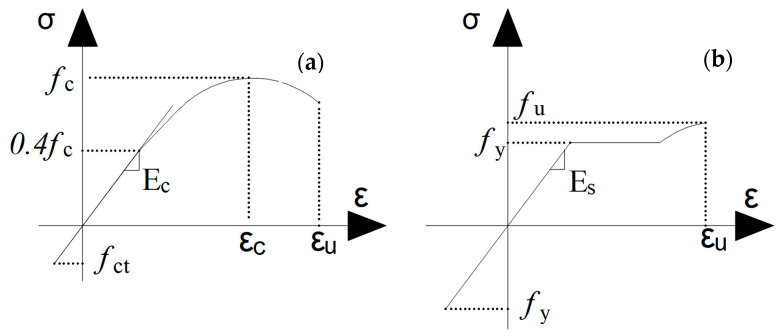
Stress-strain curves for (**a**) concrete and (**b**) steel, both adapted from [21].

This subsection is about understanding how the material properties of concrete and steel affect the behavior of reinforced concrete. For this purpose, it is important to define the stress-strain curves of these materials (Figure 4a for concrete and Figure 4b for steel); where: *f_c_*—Failure stress of compressive concrete; *f_ct_*—Failure stress of tensile concrete; *E_c_*, *E_s_*—Young’s modulus of concrete and steel; *ε_u_*—Ultimate deformation of concrete and steel; *f_u_*—Failure stress of steel, and *f_y_*—Yield stress of steel.

**Figure 5:**
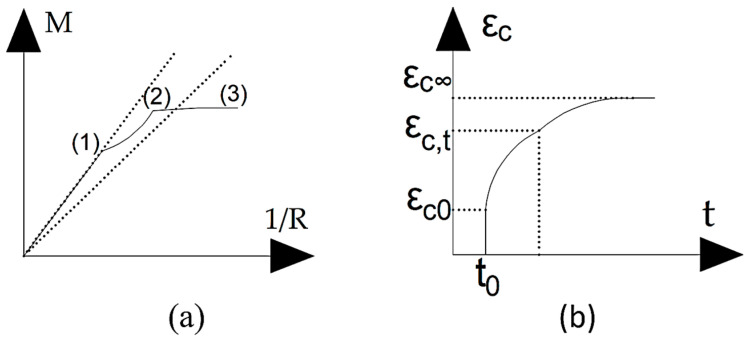
(**a**) Momentum-curvature curve of reinforced concrete, adapted from [21], (**b**) Illustrative graphic of creep, adapted from [22]. (1) non-crack mode, (2) transition mode, (3) crack mode.

Equations (6) and (7) present the constitutive model for concrete used in this work, which is taken from EC2. Figure 5a shows the momentum-curvature curve for reinforced concrete, where point 1 denotes the occurrence of cracks. Point 2 is the point at which the reinforcement reaches the yield stress, and point 3 is the failure of the structure, with straight line I representing the uncracked state and straight line II representing the cracked state.

(6) $$\begin{array}{c} \sigma_{c}<0\{\begin{array}{l} \sigma_{c}=E_{c}\varepsilon_{c}\\ \sigma_{c}=\left[1-\frac{\left(k\times {\bar{\varepsilon }}_{c}-{\bar{\varepsilon }}_{c}^{2}\right)f_{c}}{\left(1+\left(k-2\right)\times {\bar{\varepsilon }}_{c}\right)E_{c}\varepsilon_{c}}\right]E_{c}\varepsilon_{c} \end{array}\begin{array}{c} \;\;\;if\;\varepsilon_{c}\leq \frac{0.4f_{c}}{E_{c}}\\ \;\;\;if\;\varepsilon_{c}>\frac{0.4f_{c}}{E_{c}} \end{array}\\ \mathrm{With}\\ k=\frac{1.05E_{c}\varepsilon_{c}}{f_{c}} \end{array}$$

(7) $$\begin{array}{c} \sigma_{c}\geq 0\{\begin{array}{l} \sigma_{c}=E_{c}\varepsilon_{c}\\ \sigma_{c}=\left(1-d_{t}\right)E_{c}\varepsilon_{c} \end{array}\begin{array}{c} \;\;\;if\;\varepsilon_{c}\leq \frac{f_{ct}}{E_{c}}\\ \;\;\;if\;\varepsilon_{c}>\frac{f_{ct}}{E_{c}} \end{array}\\ \mathrm{With}\\ d_{t}=1-\frac{\sigma_{c}}{f_{ct}} \end{array}$$

Creep is defined according to [23] as the increase in deformation over time at constant stress. Figure 5b shows this behavior as a deformation-time curve. This phenomenon is explained by a change in the volume of water in the molecular structure in the hardened cement paste [24], which is due to factors such as duration and intensity of loading, age of the concrete at the first loading and relative humidity (EC2 [16]). The creep coefficient is the ratio between the increase in deformation and the value of the initial deformation in a given period of time. However, this coefficient is also a function of the Young’s modulus of the concrete [21]. Therefore, the creep phenomenon is taken into account when the Young’s modulus decreases with time and is calculated accordingly to EC2 [13] using Equation (8).

(8) $$E_{c,eff}=\frac{E_{cm}}{1+\varphi \left(\infty ,t_{0}\right)}$$

where: *E_cm_*—secant Young’s modulus of concrete; $\varphi \left(\infty ,t_{0}\right)$– creep coefficient in a given period of time; *ε_c_*—deformation of concrete; *ε_c__∞_*—infinite deformation of concrete; *ε_c,t_*—deformation of concrete in a given period of time (see Figure 5b). The value of the secant Young’s modulus is given in EC2. The value of the creep coefficient is determined using the estimated expressions in Annex B of EC2, taking into account as parameters the relative humidity, the age of the concrete at the time of loading in days, the equivalent thickness and the cement class of the concrete.

**Figure 6:**
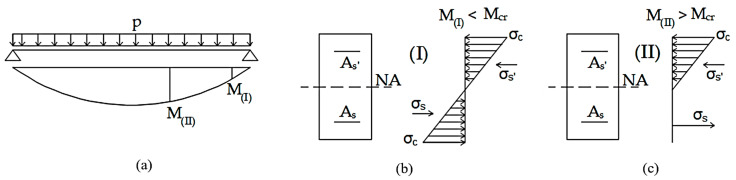
(**a**) Simply supported beam with distributed load, (**b**) cross-section stress at the state I and (**c**) at state II.

Cracking in a particular cross-section is said to occur when the most stressed fiber reaches the minimum strength and shows up as a crack in the concrete. Therefore, the tensile stress passes into the steel and causes a sudden rupture of its structural stiffness [21], as can be seen in Figure 5a. Figure 6a presents a simply supported beam with distributed load. *M_I_* is considered to be below the cracking moment (*M_cr_*), and *M_II_* is superior to this momentum. Figure 6b,c also show the stresses of concrete and steel for moments I and II (where state I is uncracked and state II is cracked). Therefore, the beam stiffness is variable and depends on whether it is uncracked or cracked. To calculate the deformations of a simply supported beam, it is necessary to make an integral by the Principle of Virtual Works (PVW), and considering all these limitations, an analytical calculation is difficult. Then, it is necessary to use computational methods.

From the above it can be seen that the difficulty of the analytical calculation is due to the value of the beam stiffness to be used, since in reinforced concrete structures it is not constant with increasing load, notably, immediately after reaching the crack in the most stressed cross-section. When analyzing the deformation of a reinforced concrete structure under a specific load, it is necessary to consider whether a cross-section will crack under that load. The curvature (1/r– where *r* is the radius) is defined in Equation (9), where the subscript *c* indicates the parameters for concrete, the subscript *I* indicates whether it is state I or II, *M* is the moment due to the actual load on the structure, and *I* is the inertia. Thus, the curvature is constant and corresponds to the curvature in state I until the crack. After cracks appear, there is then an abrupt loss of stiffness in the structure with a reduction of Young’s modulus, increasing the overall deformability of the structure. One then speaks of a curvature in the state II, which results from a cracked cross-section [21].

(9) $${\left(\frac{1}{r}\right)}_{I\;}=\frac{M}{E_{c}I_{I}}\;\;\;\mathrm{and}\;{\left(\frac{1}{r}\right)}_{II\;}=\frac{M}{E_{c}I_{II}}$$

The cracking moment is defined in Equation (10), where *f_ctm_* is the average value of the tensile strength of the concrete, *I_c_* is the inertia of the concrete, and *z* is the distance between the neutral axis and the most stressed tensile fiber.

(10) $$M_{cr}=f_{ctm}\times \frac{I_{c}}{z}$$

Finally, based on CEB [25], the long-term deformation calculated by the global coefficients method is expressed in Equation (11), where *η* is a coefficient depending on the percentage of compressive and tensile reinforcement and the Young’s modulus of steel and concrete, *k_t_* is a coefficient from [26], and *a_c_* is the deflection value.

(11) $$a_{t}={\left(\frac{h}{d}\right)}^{3}\eta \;k_{t}\;a_{c}$$

## 3. Analytical Model

**Figure 7:**
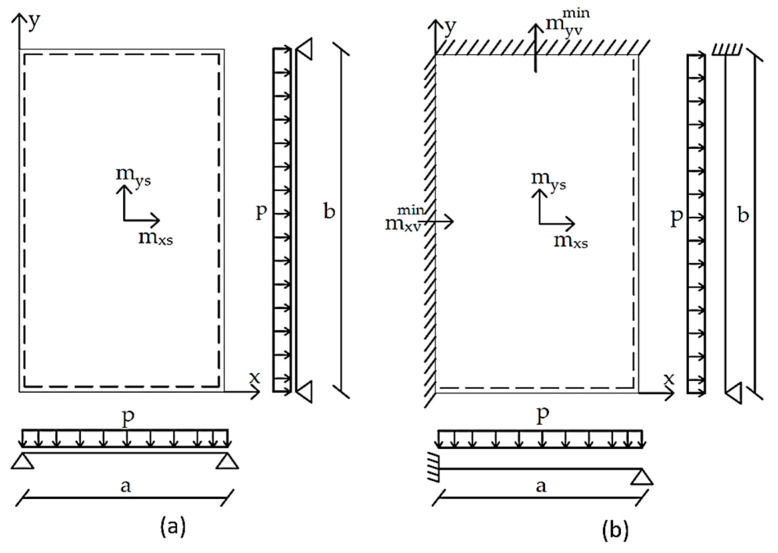
(**a**) The simply supported slab and (**b**) supported fixed-ended slab.

**Figure 8:**
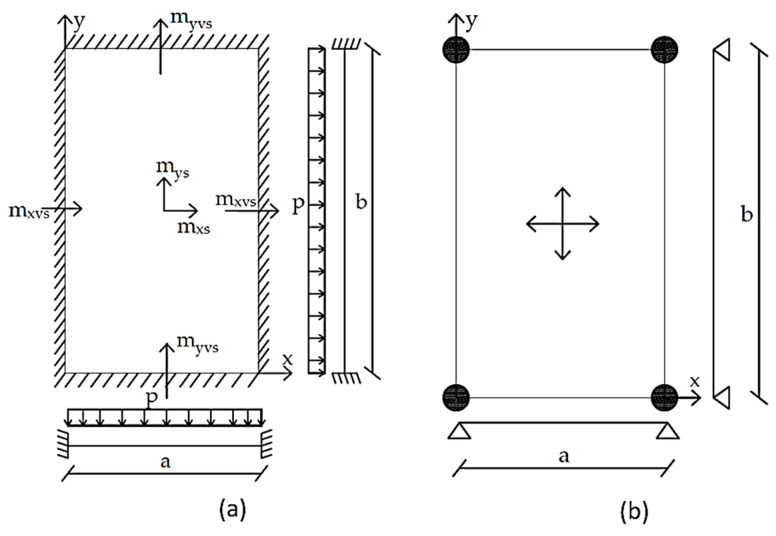
(**a**) Fixed-ended slab and (**b**) flat slab.

For the calculation of deflections in slabs, Barés [27] tables were used. For the calculation of deformation and internal forces (bending moments mys, mxs, myv, mxv), the following hypotheses were assumed: (i) physical linearity, (ii) geometric linearity, (iii) material homogeneity, and (iv) Kirchoff hypotheses—the normal fibers in the midplane of the slab remain undeformable and are perpendicular to the midplane after deformation. The solution of the equations that determine the structural behavior of the slabs is very complex. Therefore, numerical methods are used [27]: double series and finite differences. According to Section 2, the representation of the slabs for different parameterizations are displayed in Figure 7 and Figure 8. All Barés [27] tables can be consulted in Annex B of [28].

To obtain the deflection of slabs, the coefficient *γ* (Equation (12)) must be determined [26], where the parameters *a* and *b* are the spans of the slab, and the coefficient varies between 0.5 to 2.0, since outside this range the slab is considered to have a cylindrical bending behavior. Finally, the deflection value of the slab is given in Equation (13) [26].

(12) $$\gamma =\frac{a}{b}$$

(13) $$a_{c}=k_{bares}\frac{pa^{4}}{E\;h^{3}}$$

On the other hand, the deflection of a beam is calculated using the PVW, which has the mathematical formulation below [29] (Equation (14)), where the subscript *l* indicates that it is an integral over the length of the element, 1/r is the curvature (see Equation (9)) and *M’* is the momentum at that point due to a unit load with the direction at the point of displacement to be calculated. Thus, to calculate the deformation of the element, one must determine its curvature. Although it is a simple estimate, the deflection value is degraded because it is not very representative of reality. It therefore does not take into account factors relevant to the deformability of reinforced concrete, such as the influence of reinforced cracks and long-term effects such as creep and retraction.

(14) $$a=\int_{l}\frac{1}{r}M^{\prime }dx$$

**Figure 9:**
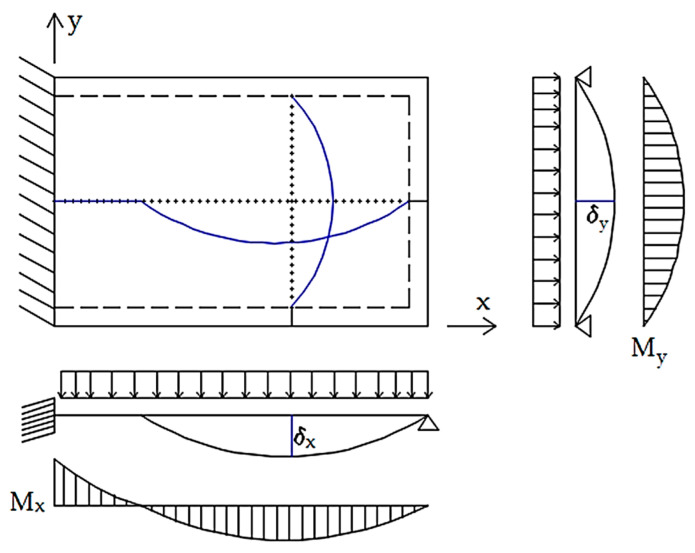
Displacement compatibility at one point for a slab with one fixed-ended border.

**Figure 10:**
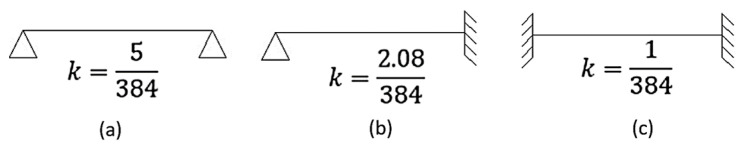
*k* coefficient for (**a**) simply supported slab, (**b**) supported fixed-ended slab and (**c**) fixed-ended slab.

Finally, the distribution of the slab loads among the beams was made by the bands method, assuming that the load in the slab can be balanced by displacement, which ensures compatibility at one point of the slab. In this method, the slab is divided into strips in the *x* and *y* directions, and each strip is examined in only one direction, according to [30]. Since the distribution of loads depends on the support stiffness and the span values, a correct load path is required. Figure 9 shows an example of a slab with one edge fixed and the rest simply supported, using a band in each direction, which is used in this work. Equation (15) is used to calculate the displacement of each of these bands [31].

(15) $$\delta_{i}=\frac{k_{i}\times q_{i}\times l_{i}^{4}}{EI}\;\;\;\;i\in \left[x;y\right]$$

where: *k_i_*—band stiffness depending on support condition in *i* direction; *q_i_*—load in *i* direction; *l_i_*—span in *i* direction. The coefficient *k* depends on the support conditions of the slab; the values are shown in Figure 10. To ensure that the displacement of the slabs occurs at the same point, the expression of the displacement is balanced in both directions ($\delta_{x}=\delta_{y}$). This is an equilibrium condition for the slabs, where the sum of the loads in both directions is equal to the load acting on the slab ($q=q_{x}+q_{y}$) [32]. By replacing the previous equations with another, one can calculate the value of the distributed load in one direction (Equation (16)).

(16) $$q_{i}=q\times \frac{1}{\frac{k_{i}\times l_{i}^{4}}{k_{j}\times l_{j}^{4}}+1}\;,\;i,j\in \left[x;y\right]$$

## 4. Numerical Model

The materials to be defined in the finite element model in SAP2000 are steel and concrete. To achieve this, it is necessary to analyze their isotropy and non-linearity. The numerical model for the definition of concrete uses isotropic material definition, and the steel was uniaxial material. The material non-linearity is considered with the directional model. Thus, several stress-strain curves are modelled for one or more components. In this work, a Shell element (with 6 degrees of freedom in each node) was used to model slabs and beams to obtain more accurate results. Furthermore, a thick slab formulation (Mindlin/Reissner) [33], referred to as shell-thick in SAP2000, was used to model the structural behavior of the slab. With this formulation, better results can be obtained in areas with high stress concentrations [34].

**Figure 11:**
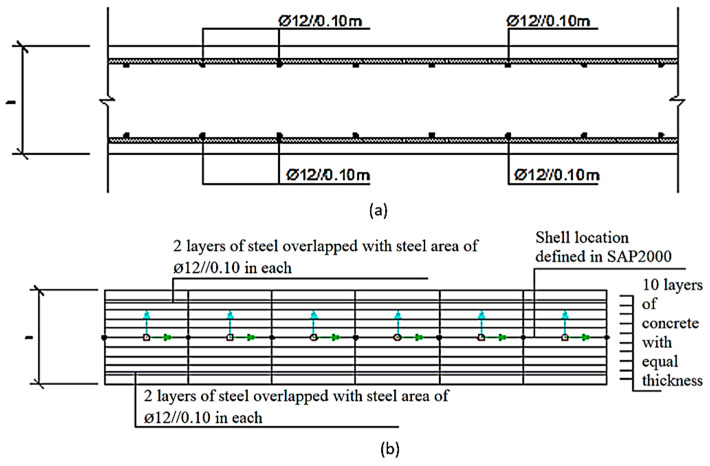
Cross-section for (**a**) Reinforced concrete slab and (**b**) non-linear Shell element.

**Figure 12:**
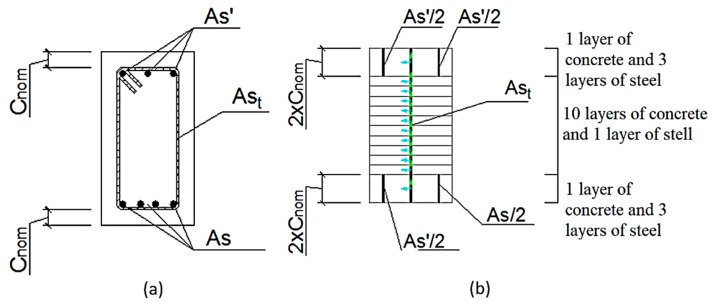
Cross-section for (**a**) Reinforced concrete beam and (**b**) non-linear Shell element.

The multi-layer method is a heterogeneous cross-section designed along the thickness of the shell by independent layers. In the heterogeneous cross-section, it is possible to consider the non-linearity of the material. The schematics of the slab and the beam are illustrated in Figure 11 and Figure 12, respectively. Further details on the material directional model can be found in [35].

**Figure 13:**
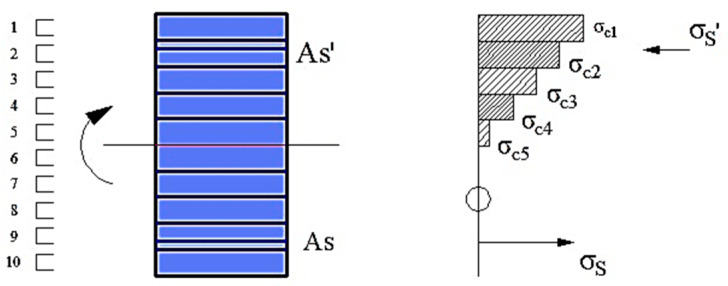
Stress distribution in a cracked RC cross-section with 10 layers in SAP 2000.

It is appropriate to define a high number of layers, because in this way the correct stress distribution in the reinforced concrete cross-section is achieved. In SAP2000, it is possible to define a stress-strain curve of the material used, which makes it possible to model a cracked cross-section, as shown in Figure 13.

## 5. Parametric Campaign

The parametric study developed aims mainly to evaluate the deformations previously described by analytical and numerical models. Three different cases have been developed, the difference being in the behavior adopted for the different materials. In the different models, the materials used are always the same, but with different stress-strain curves. For the concrete, C30/37 was chosen because it is a commonplace type whose characteristic compressive strength (*f_ck_*), average tensile strength (*f_ctm_*), and Young’s modulus (*E_c,m_*) after twenty-eight days are described in Table 2. The steel chosen was A500NR, whose characteristic yield strength (*f_yk_*) and Young’s modulus (*E_s_*) are listed in Table 3.

**Figure 14:**
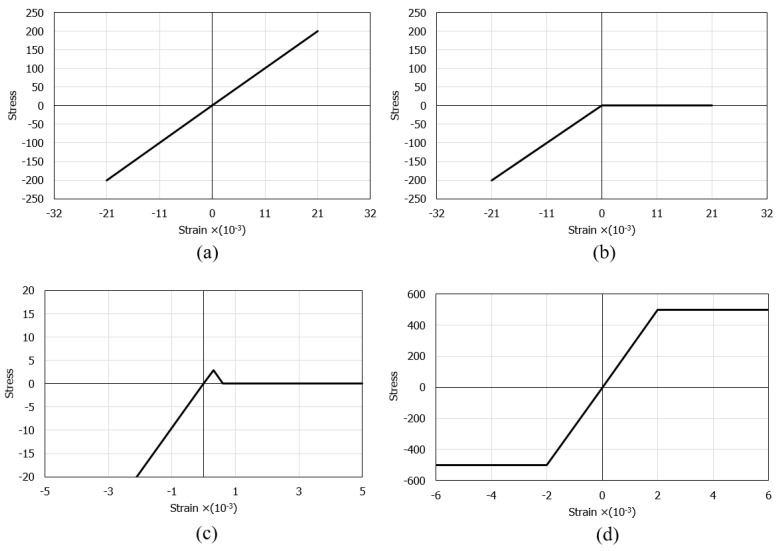
Stress-strain curves for (**a**) LM; (**b**) 1NLM; (**c**) 2NLM and (**d**) steel used in SAP2000 (values in MPa and m/m).

The cases were divided into a linear model and two non-linear models. The linear model (LM) admits that the cross-sections are made exclusively of concrete with linear elastic behavior in both compression and tension, showing the stress-strain curve (Figure 14a) with a slope equal to Young´s modulus of the concrete under consideration. The first non-linear model (1NLM) admits that the concrete has zero tensile strength, and the compressions behave similarly to the LM (Figure 14b). The second non-linear model (2NLM) admits that the concrete has a maximum tensile strength of 2.9 MPa (Figure 14c). The steel exhibits the same stress-strain curve in both non-linear models (Figure 14d).

**Figure 15:**
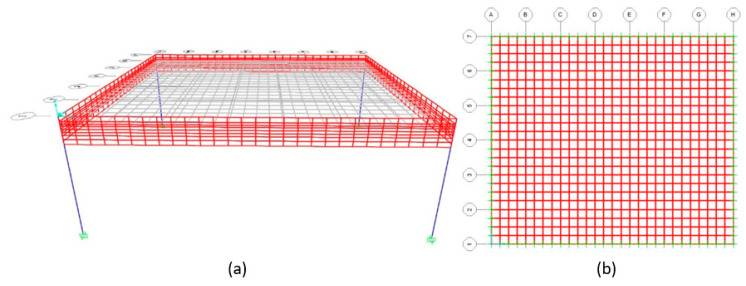
Finite element meshes for (**a**) beam and (**b**) slab models.

**Figure 16:**
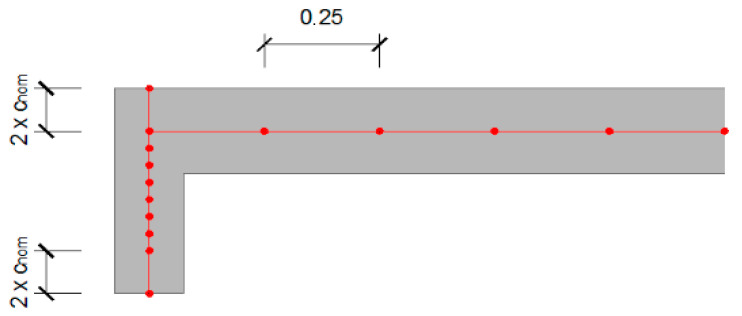
Connection scheme for slab with the beam in the finite element model.

Figure 15 represents the finite element meshes used for the different structural elements analyzed. For the slab model, the finite element mesh consists of 0.25 m square elements. For the beam model, the mesh consisted of 0.25 m square elements and was designed as described in the previous section. The mesh in which the beam reinforcement is inserted has a sectional dimension of 0.1 m (twice the size of the coating), and the remaining area was divided into elements with smaller dimensions. The connection scheme for the slab with the beam is shown in Figure 16.

**Figure 17:**
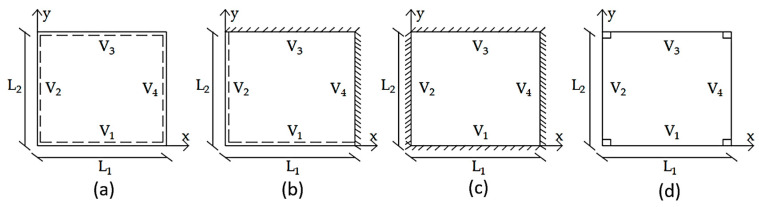
Scheme for (**a**) simply supported slab; (**b**) fixed-ended supported slab; (**c**) fixed-ended slab and (**d**) flat slab.

A uniform load of 10 kN/m^2^ was applied to the slab to analyze the deformations of the structural elements. It is worth mentioning that this load is close to the permanent combination of loads (dead weight of the slab, permanent load and characteristic live loads) for the current residential pavement uses. In order to study the differences between the deformations determined from analytical and numerical models, the ratio (*r =*$\;\delta_{numerical}/\delta_{analytical}$) of the deformations is calculated. The deformation limit values determined according to EC2 and REBAP, respectively, are: δ<l/400 and δ<l/250. The graphs in this section refer to square parameterizations (*l_1_ = l_2_*; see Figure 17). All others cases (*l_1_* and *l*_2_ vary from 4m to 8m) can be found in annex D of [28]. Finally, Figure 17 shows the schematic of all slabs used in this parameterization study.

### 5.1. Simply Supported Slab

It is challenging to find a slab designed to be simply supported (Figure 17a). However, since it is the simplest geometry for analytical calculation and therefore gives better results, it was also chosen for finite element modelling to compare the results. The thickness of the slab was calculated as a function of the smallest span and for the different parameterizations. These values are shown in Figure 1a. The values of the analytical and numerical deformations can be found in Tables C.1 and C.2, respectively, in annex C of [28].

**Figure 18:**
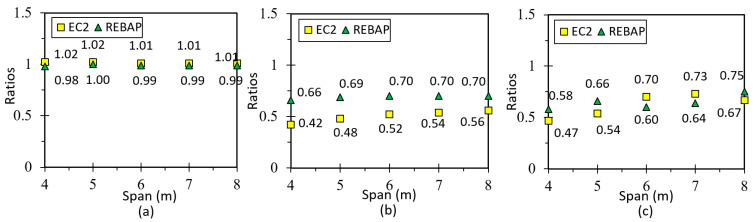
Ratios values for (**a**) LM, (**b**) 1NLM and (**c**) 2NLM, for simply supported slab.

The following figure shows the ratios (*r*) of the analytical and numerical values of the simply supported slab designed according to EC2 and REBAP for LM. Observation of Figure 18a suggests that the *r* values are close to 1.0, so the LM is valid. The remaining graphs for this type of slab are in annex D1 of [28], and the values of the deformation ratio are approximated to 1.0. Figure 18b,c show the results of the ratios for non-linear models. It can be noted that the deformation values obtained by SAP2000 are lower than the values obtained by analytical calculation, because they have significant differences in the calculation methodology.

**Figure 19:**
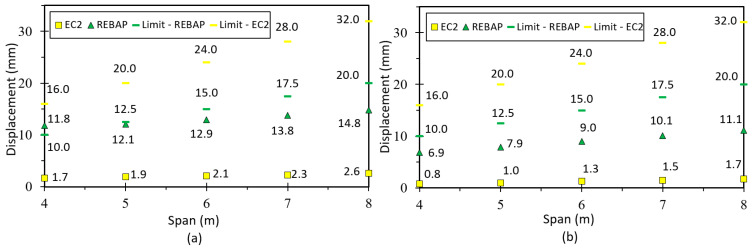
Deformation values of 2NLM: (**a**) analytical and (**b**) numerical, with their limit values, for simply supported slab.

The limit values of deformation are shown in Figure 19. It can be observed that for the square parameterizations, the deformation values are always lower than the limit values. The deformations calculated with the EC2 are always below the limit values for all parameterizations. However, the parameterizations 5 × 4 m, 6 × 4 m, 7 × 5 m, 8 × 5 m and 8 × 6 m designed according to REBAP do not comply with their limit values. The 7 × 4 m and 8 × 4 m parameterizations do not respect both limit values. The analytical deformation of REBAP for rectangular parameterizations has never kept its limit values.

### 5.2. Fixed-ended Supported Slab

**Figure 20:**
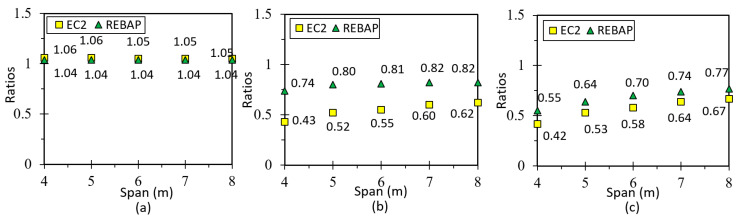
Ratios values for (**a**) LM, (**b**) 1NLM and (**c**) 2NLM, for fixed-ended supported slab.

This geometry is used to obtain the deformation of a corner slab panel (Figure 17b). The minimum thickness values calculated according to REBAP and EC2 are displayed in Figure 2a. The values of analytical and numerical deformations can be found in Annex 3, Tables C3 and C4, respectively, of [28]. The ratios between analytical and numerical values are displayed in Figure 20a for LM and Figure 20b,c for non-linear models. The ratio values for LM are approximated to 1.0, which means that the deformations calculated by the analytical and numerical methods are almost identical. However, as in the case of the simply supported slab for non-linear models, the ratio value is lower than 1.0. Thus, the numerical values are lower than those of the analytical calculation.

**Figure 21:**
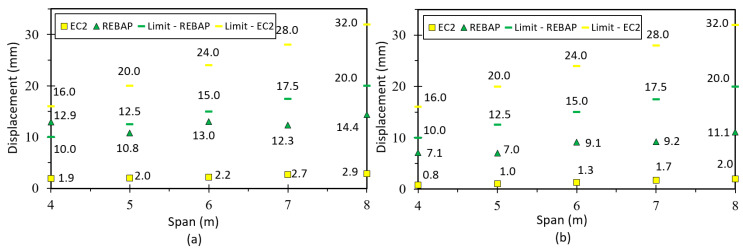
Deformation values of 2NLM: (**a**) analytical and (**b**) numerical, with their limit values, for fixed-ended supported slab.

Comparing the limit values (Figure 21a,b), it can be seen that the analytical deformation values are higher than the numerical ones. It can also be noted that for the 4 × 4 m parameterization designed according to REBAP, the analytical deformation is higher than the limit value for REBAP. However, this limit value is respected for the numerically calculated deformation. The limit values designed according to EC2 are always respected. However, the numerical deformations of the 2NLM for the 5 × 4 m, 6 × 4 m, 7 × 5 m, 8 × 5 m and 8 × 6 m parameterizations do not comply with the limit values of REBAP. In addition, the 7 × 4 m and 8 × 4 m do not comply with any limit values.

### 5.3. Fixed-Ended Slab

**Figure 22:**
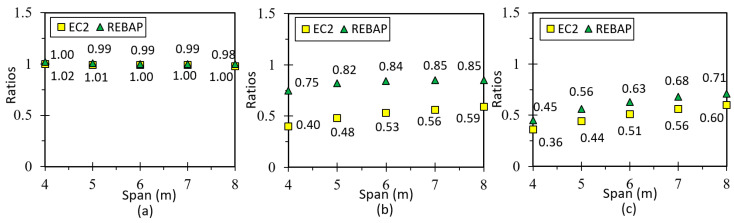
Ratios values for (**a**) LM, (**b**) 1NLM and (**c**) 2NLM, for fixed-ended slab.

This parameterization is intended to simulate the behavior of an interior slab panel (Figure 17c). The minimum thickness depends on the smallest span and can be consulted in Figure 2b. All values of analytical and numerical deformations can be found in the work of [28] Annex C, Tables C5 and C6, respectively. Figure 22a presents the results for the ratio for LM, which is close to 1.0 as in the other cases, suggesting that it is validated. In the graphs of Figure 22b,c, the ratios of non-linear deformations are observed. As in the previous cases, it can be seen that the deformation values obtained with SAP2000 are lower than those resulting from the analytical calculations.

**Figure 23:**
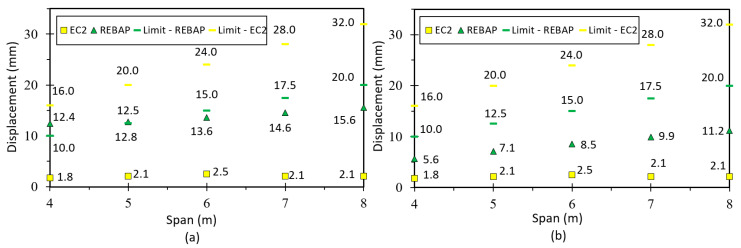
Deformation values of 2NLM: (**a**) analytical and (**b**) numerical, with their limit values, for fixed-ended slab.

Comparing the limit values (Figure 23a,b), it can be observed that the analytical deformation values are higher than the numerical ones for REBAP, although the results for EC2 are the same. It is verified that for square parameterizations the values of numerical deformation are lower than the limit values. However, for the 4 × 4 m and 5 × 5 m parameterizations, the analytical deformation values according to REBAP are higher than their limit values. The deformation values calculated according to EC2 are never exceeded, regardless of the evaluation of the limit values.

### 5.4. Flat Slab

This type of slab is used to compare the analytical results with the finite element model (Figure 17d). Determining the deformation for this geometry by the known analytical means only allowing the square slab to be studied. The minimum thickness depends on the smallest span and can be consulted in Figure 3. All values of analytical and numerical deformations can be found in [28] Annex C, Tables C7 and C8, respectively.

**Figure 24:**
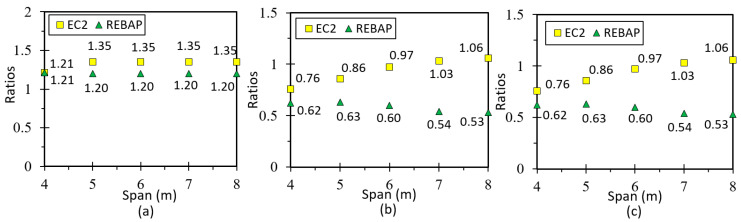
Ratios values for (**a**) LM, (**b**) 1NLM and (**c**) 2NLM, for flat slab.

The results of LM are presented in Figure 24a, with values higher than 1.0 because of uncertainty in the analytical expression used to calculate the deformation in the flat slab. In addition, the support conditions of the analytical model are different from those of the numerical model. Interpretation of Figure 24b,c shows that the ratio of deformations for both non-linear models is close to 1.0 for EC2. On the other hand, for REBAP, the analytical deformation is superior to the numerical one, due to the same reasons as for LM.

**Figure 25:**
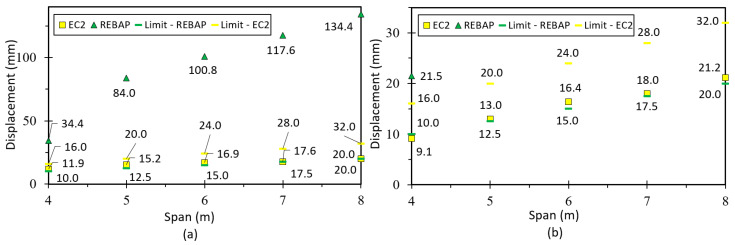
Deformation values of 2NLM: (**a**) analytical and (**b**) numerical, with their limit values, for flat slab.

In the case of a flat slab, the deformation is higher than for the other types of slabs. However, by observing both graphs (Figure 25), it can be concluded that the deformations for flat slabs dimensioned according to REBAP are higher than the limit values of the two structural codes. The deformation of EC2 is higher than the limit imposed by REBAP, but does not exceed its limit.

### 5.5. Supported Beam

In order to correctly model the simply supported beam, the model under study assumes that the beams support a panel slab, according to Figure 17a. The span dimensions of the beams also vary between 4 m and 8 m, and the heights used are shown in Figure 1b. All values of analytical and numerical deformations are given in Annex C, Tables C9 and C10, respectively, of [28].

**Figure 26:**
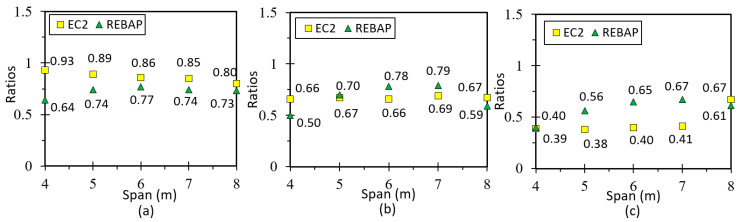
Ratios values for (**a**) LM, (**b**) 1NLM and (**c**) 2NLM, for supported beam.

The results from LM are presented in Figure 26a. These results reveal a greater inaccuracy compared to the values obtained for the slabs, which can be explained by the uncertainty of the transmitted loads of the slab and may be related to the value of the inertia to be used concerning the use of the slab “shear lag effect” [36]. For the non-linear models (Figure 26b,c), the values of the analytical deformation are higher than the numerical values in most cases.

**Figure 27:**
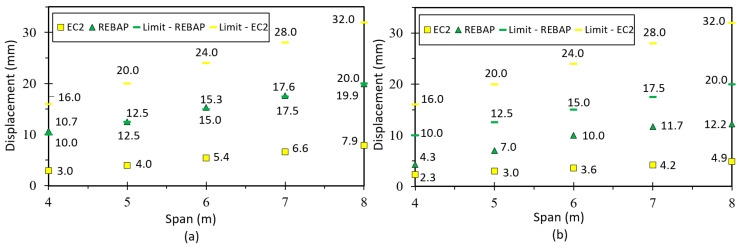
Deformation values of 2NLM: (**a**) analytical and (**b**) numerical, with their limit values, for supported beam.

By observing Figure 27, it can be verified that the numerical deformation values are lower than the limit values. However, the values of analytical deformation are higher or identical to the limit values of REBAP.

## 6. Case Study

This section analyzes three cases of the study of floors with different typologies. In each case, three models are considered, namely LM, 1NLM and 2NLM, explained in the previous section. The same material was used in all models, as described in Table 2 and Table 3. The reinforcement of the concrete was 1.5% for the beams and 0.5% for the slabs, but for the flat slab, the exact value of the beams was used in areas close to the columns. The thickness of the slab is constant throughout the floor and corresponds to the highest calculated thickness value for each slab panel. The same dimensions were adopted for continuous beams. The loads applied in the slab were increased by the following combinations: Almost Permanent Combination (APC) and Frequent Combination (FC), which can be seen in Equations (17) and (18), respectively. The results presented in this section refer to the most severe case of deformation.

(17) $$E_{d}=\underset{j\geq 1}{\sum }G_{k,j}+P\;+\underset{i>1}{\sum }\psi_{2i}Q_{k,i}$$

(18) $$E_{d}=\underset{j\geq 1}{\sum }G_{k,j}+P+\psi_{1,1}Q_{k,1}+\underset{i>1}{\sum }\psi_{2,i}Q_{k,i}$$

### 6.1. Housing Floor

**Figure 28:**
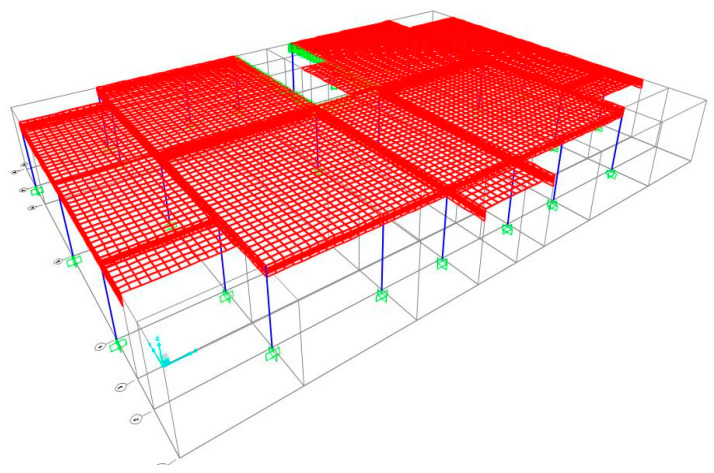
Finite element mesh of the first case of study.

The first study case deals with a fixed-ended slab of a housing floor. The architectural blueprint can be found in Annex E1 of [28]. The arrangement of the structural elements can be found in the design blueprint (Annex E2 of [28]). The finite element model used in this case is shown in Figure 28. The loads considered in this case are: (i) dead weight of the slab; (ii) dead weight of the beam; (iii) dead weight of the partition walls (2.0 kN/m^2^) [26]; (iv) permanent load (1.5 kN/m^2^) [26] and (v) overload [16] (2.0 kN/m^2^). Slabs and beams are designed according to Section 2.1 and Section 2.2. Since $\rho >\rho_{0}$, Equation (4) is applied, and thus $\frac{l}{d}=18,2≅18$. The beam height is 0.26 m (Equation (19)), and the thickness of the slab is 0.23 m, with materials properties in Table 4.

(19) $$h_{beam}=\frac{3.8}{18}+0.05=0.26m$$

According to the EC2 and REBAP pre-design criteria, the dimensions of the structural elements are given in Table 5 and Table 6.

The results of LM are shown in Table 7 as numerical and analytical values ratios. The calculation of the analytical deformation of the slabs was performed using tables of Bare´s in Annex B (Figures B1 and B4) of [28]. Therefore, the ratio values for LM and 2NLM are lower than 1.0 because the analytical deformations are higher than the numerical ones.

By observation of Table 8, the deformation values are lower than the limit values, regardless of the type of analysis performed. It is noted that for 2NLM, the numerical values are lower. The values of 1NLM are higher than those of 2NLM; therefore, it was more unfavorable.

### 6.2. Flat Slab Housing Floor

**Figure 29:**
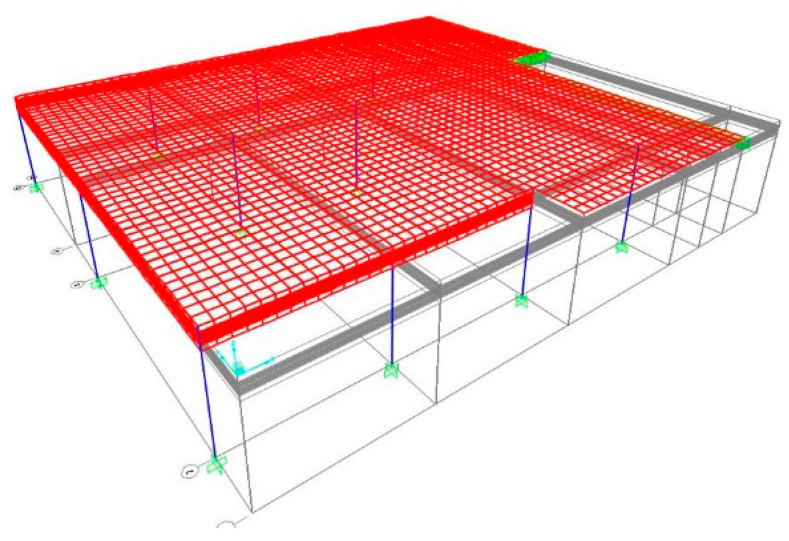
Finite element mesh of the second case of study.

The second study case is a housing floor with a flat slab supported on beams. The architectural blueprint can be found in Annex E3 of [28]. The arrangement of structural elements is included in the design blueprint (Annex E4 of [28]). The finite element model used in this case is shown in Figure 29, where only half of the structure was modelled to reduce processing time since it has an axis of symmetry. The load applied is the same as in the previous case because it is also a housing floor.

The beam height selected in this case is higher than the minimum height required by the respective structural code. Thus, these results are not presented. Another factor contributing to the failure to account for beam deformation is the difficulty in analytically estimating the load acting on the beam. The slab thickness according to EC2 and REBAP are shown in Table 9. The slab panel with the most severe deformation is the L3. Hence, according to the two structural codes, there is a minimum difference in thickness between the first and the second case of study.

Table 10 presents the ratio between analytical and numerical values for the second study case for L3. The calculation of the analytical deformation of the slabs was performed using the tables of Bare´s in Annex B (Figures B6 and B7) of [28]. The results presented in Table 10 are approximated to 1.0 for LM and are thus validated. Furthermore, similar to the parameterization study, the deformation ratio is found to be less than 1.0 for both non-linear models, suggesting that the values calculated using SAP2000 are inferior to the analytical ones.

Table 11 exhibits that the numerical deformation values are much lower than the analytical values. It can also be seen that the analytically calculated deformation value for the slab designed with REBAP is higher than the limit value of both combinations. The deformation of the slab calculated to EC2 is always lower than both limit values.

### 6.3. Flat Slab Garage Floor

**Figure 30:**
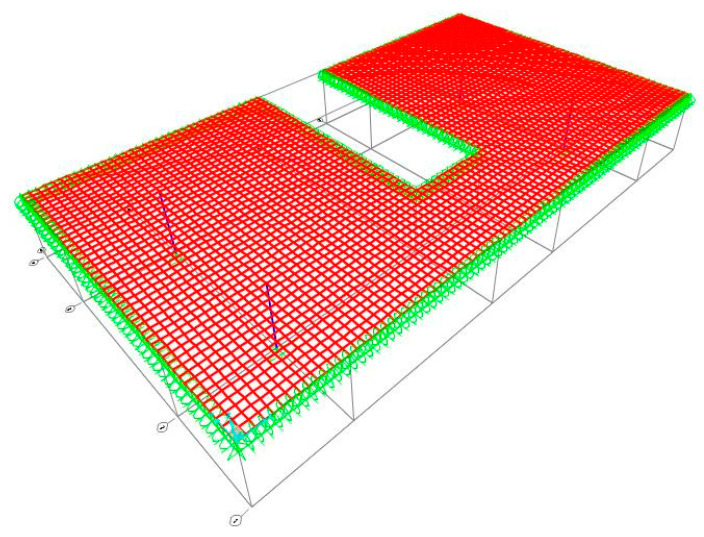
Finite element mesh of the third case of study.

The last study case is a garage floor with a flat slab supported by structural walls at the edges and columns on the interior. The architectural blueprint can be found in Annex E5 of [28]. The layout of the structural elements can be found in the design blueprint (Annex E6 of [28]). Figure 30 shows the finite element model used in this study case. The loads considered in this case are: (i) dead weight of the slab; (ii) permanent load (1.5 kN/m^2^) [26] and (iii) overload [16] (2.5 kN/m^2^).

The design values of the slab in this case of study are shown in Table 12. As with the previous case, analytical deformation of the slabs was performed using the tables of Bare´s in Annex B (Figures B6 and B7) of [28]. The slab panel where the deformation is the most severe is the L4. In the case of a flat slab, the minimum difference thickness is higher than in the first case of study and similar to the second case.

Table 13 presents the ratio between analytical and numerical values for the third study case for L4. The analytical deformation calculation was performed in a similar way as for the previous cases using the same tables. It is found that the values of analytical deformation for EC2 are lower than the numerical values for LM. Furthermore, it is verified that the deformation ratio for both non-linear models is significantly lower than 1.0, which suggests that the values calculated using SAP2000 are inferior to the analytical values, with the values calculated using REBAP being the lowest.

Table 14 presents that the numerical deformation values for both combinations are much lower than the analytical values. The deformation of the slab designed according to EC2 is always lower than both limit values. On the other hand, the analytically calculated deformation values are much higher than the numerical values, according to REBAP. It can be observed that the analytical value for FC is higher than the limit value for REBAP.

## 7. Conclusions

This work’s main objective was to numerically simulate the deformation of reinforced concrete slabs and beams according to the slenderness criteria of EC2 and REBAP. The numerical models were based in finite element shells with layers of reinforced concrete material, providing non-linear analysis. Validation was performed using known analytical expressions and long-term deformations with CEB [25]. The non-linear behavior for steel and concrete was considered. The main conclusions of this work are: (i) the values of long-term deformation of slabs using analytical methods are very conservative and uneconomical since they rely on solutions with thicker concrete cross-sections; (ii) the indirect deformation of REBAP is underestimated for beams and in some cases for slabs; (iii) the current verification of deformation, based on simplified criteria, has some limitations, especially for rectangular slabs, due to the lack of knowledge of the non-linear distribution of loads along the beams; (iv) the 2NLM is advantageous because it uses the tensile strength of the concrete, which is important when considering the minimum slenderness of the EC2 because the CEB are too conservative for non-linear effects unlike the numerical models; (v) the EC2 has some inconsistencies in the design of simply supported slabs because it requires a greater thickness than a flat square slab. With these numerical non-linear tools it is possible to reduce the thickness of concrete cross sections, and provide a more economical solution, in terms of controlling the maximum allowed deflection.

## Figures and Tables

**Table 1:** Ratio values (span/height) for indirect deformation control in REBAP.

Structural Element	*l/h*
Two-way reinforced simply supported slab	34
Two-way reinforced supported fixed-ended slab	40
Flat slab	34
Two-way reinforced fixed-ended slab	48
Simple beam	16
Two-way supported beam	20
Fixed-ended beam	27

**Table 2:** Concrete C30/37 properties.

*f_ck_* (MPa)	*f_ctm_* (MPa)	*E_c,m_* (MPa)
30.0	2.9	33.0

**Table 3:** Steel A500NR properties.

*f_yk_* (MPa)	*E_s_* (MPa)
500	200

**Table 4:** Data to calculate beam height to verify indirect deformation, according to EC2.

*l* [m]	*K*	*ρ*	*ρ’*	*ρ_0_*	*f_ck_* (MPa)
3.8	1.3	0.015	0	0.0055	30.0

**Table 5:** Beams dimension for first case of study (*h*, *b*, *As*, *As’* values in m and cm^2^).

EC2	*h*	*b*	*A_s_*	*A_s_’*	REBAP	*h*	*b*	*A_s_*	*A_s_’*
V1	0.34	0.15	6.53	6.53	V1	0.26	0.15	4.73	4.73
V2	0.44	0.18	8.78	8.78	V2	0.35	0.15	6.75	6.75
V3	0.38	015	7.43	7.43	V3	0.30	0.15	5.63	5.63

**Table 6:** Slab dimension for first case of study (*e*, *As*, *As’* values in m and cm^2^/m).

Slab	*e*	*A_s_*	*A_s’_*
EC2	0.23	4.73	4.73
REBAP	0.20	8.50	8.50

**Table 7:** Deformation ratios from LM, 1NLM and 2NLM for the first case of study.

Linear Model					1° Non-Linear Model				2° Non-Linear Model			
	EC2		REBAP		EC2		REBAP		EC2		REBAP	
Comb.	APC	FC	APC	FC	APC	FC	APC	FC	APC	FC	APC	FC
Beam	0.93	0.92	1.04	1.04	1.27	1.25	1.77	1.76	0.81	0.79	0.81	0.81
Slab	0.79	0.76	0.63	0.63	0.57	0.56	0.54	0.53	0.49	0.50	0.46	0.41

**Table 8:** Deformation and limit values of the 2NLM for the first case of study (values in mm).

Comb.	APC			FC				APC			FC		
EC2	δ_Analy_	δ_Num_	δ_limit_	δ_Analy_	δ_Num_	δ_limit_	REBAP	δ_Analy_	δ_Num_	δ_limit_	δ_Analy_	δ_Num_	δ_limit_
Beam	5.39	4.34	24	5.69	4.51	15	Beam	8.46	6.88	24	8.83	7.16	15
Slab	6.52	3.21	24	6.80	3.42	15	Slab	9.19	4.27	24	10.89	4.46	15

**Table 9:** Slab dimension for second case of study (*e*, *As*, *As’* values in m and cm^2^/m).

Slab	*e*	*A_s_*	*A_s’_*
EC2	0.28	12.5	12.5
REBAP	0.20	10.0	10.0

**Table 10:** Deformation ratios from LM, 1NLM and 2NLM for the second case of study of L3.

L3	Linear Model		1° Non-Linear Model		2° Non-Linear Model	
Combination	APC	FC	APC	FC	APC	FC
EC2	1.2	1.2	0.86	0.86	0.66	0.66
REBAP	0.9	0.9	0.75	0.76	0.4	0.4

**Table 11:** Deformation and limit values of the 2NLM for the second case of study (values in mm).

Comb.	APC			FC		
	δ_Analy_	δ_Num_	δ_limit_	δ_Analy_	δ_Num_	δ_limit_
EC2	5.6	3.71	19.2	5.8	3.83	12
REBAP	20.7	8.31	19.2	21.5	8.66	12

**Table 12:** Slab dimension for third case of study (*e*, *As*, *As’* values in m and cm^2^/m).

Slab	*e*	*A_s_*	*A_s’_*
EC2	0.27	12.0	12.0
REBAP	0.19	8.0	8.0

**Table 13:** Deformation ratios from LM, 1NLM and 2NLM for the third case of study of L3.

L4	Linear Model		1° Non-Linear Model		2° Non-Linear Model	
Combination	APC	FC	APC	FC	APC	FC
EC2	1.4	1.3	0.64	0.63	0.41	0.41
REBAP	0.8	0.8	0.46	0.47	0.24	0.24

**Table 14:** Deformation and limit values of the 2NLM for the third case of study (values in mm).

Comb.	APC			FC		
	δ_Analy_	δ_Num_	δ_limit_	δ_Analy_	δ_Num_	δ_limit_
EC2	5.15	2.11	22	5.4	2.2	14
REBAP	20.57	4.91	22	21.7	5.3	14

## Data Availability

Not applicable.
